# Prevalence of immune-related adverse events and anti-tumor efficacy following immune checkpoint inhibitor therapy in Japanese patients with various solid tumors

**DOI:** 10.1186/s12885-022-10327-7

**Published:** 2022-11-29

**Authors:** Yuki Yoshikawa, Michio Imamura, Masami Yamauchi, C. Nelson Hayes, Hiroshi Aikata, Wataru Okamoto, Yoshihiro Miyata, Morihito Okada, Noboru Hattori, Kazuhiko Sugiyama, Yukio Yoshioka, Shigeaki Toratani, Masaaki Takechi, Tatsuo Ichinohe, Tsutomu Ueda, Sachio Takeno, Tsuyoshi Kobayashi, Hideki Ohdan, Jun Teishima, Michihiro Hide, Yasushi Nagata, Yoshiki Kudo, Koji Iida, Kazuaki Chayama

**Affiliations:** 1 Department of Gastroenterology, Graduate School of Biomedical and Health Science, Hiroshima University, 1-2-3 Kasumi, Minami-ku, Hiroshima, 734-8551 Japan; 2 Research Center for Hepatology and Gastroenterology, Hiroshima University, Hiroshima, Japan; 3 Cancer Treatment Center, Hiroshima University Hospital, Hiroshima, Japan; 4 Department of Surgical Oncology, Hiroshima University, Hiroshima, Japan; 5 Department of Molecular and Internal Medicine, Graduate School of Biomedical and Health Sciences, Hiroshima University, Hiroshima, Japan; 6 Department of Clinical Oncology, Hiroshima University Hospital, Hiroshima, Japan; 7 Department of Molecular Oral Medicine and Maxillofacial Surgery, Graduate School of Biomedical and Health Science, Hiroshima University, Hiroshima, Japan; 8 Department of Oral and Maxillofacial Surgery, Program of Dentistry, Graduate School of Biomedical and Health Sciences, Hiroshima University, Hiroshima, Japan; 9 Department of Hematology and Oncology, Research Institute for Radiation Biology and Medicine, Hiroshima University, Hiroshima, Japan; 10 Department of Otorhinolaryngology, Head and Neck Surgery, Graduate School of Biomedical and Health Sciences, Hiroshima University, Hiroshima, Japan; 11 Department of Gastroenterological and Transplant Surgery, Graduate School of Biomedical and Health Science, Hiroshima University, Hiroshima, Japan; 12 Department of Urology, Graduate School of Biomedical and Health Sciences, Hiroshima University, Hiroshima, Japan; 13 Department of Dermatology, Graduate School of Biomedical and Sciences, Hiroshima University, Hiroshima, Japan; 14 Department of Radiation Oncology, Hiroshima University Hospital, Hiroshima, Japan; 15 Department of Obstetrics and Gynecology, Graduate School of Biomedical Sciences, Hiroshima University, Hiroshima, Japan; 16 Department of Neurosurgery, Graduate School of Biomedical and Health Sciences, Hiroshima University, Hiroshima, Japan; 17 Collaborative Research Laboratory of Medical Innovation, Graduate School of Biomedical and Health Science, Hiroshima University, Hiroshima, Japan; 18 Hiroshima Institute of Life Sciences, Hiroshima, Japan

**Keywords:** Immune checkpoint inhibitors, Immune-related adverse events, Immune-related liver injury, Programmed death 1, Cytotoxic T-lymphocyte antigen 4, Combination therapy, Eosinophil count

## Abstract

**Background:**

While immune checkpoint inhibitors (ICIs) occasionally cause immune-related adverse events (irAEs) in various organs, the prevalence of irAEs and potential risk factors have not been clarified. We identified irAE predictive factors and examined the relationship between the effect of ICIs and irAEs for patients with malignancies.

**Methods:**

A total of 533 cases treated with ICIs, including programmed death 1 (PD-1), PD-ligand 1 (PD-L1), and cytotoxic T-lymphocyte antigen 4 (CTLA-4), for various malignancies were included retrospectively. We recorded irAEs from medical records and graded them using the Common Terminology Criteria for Adverse Events version 5. Prevalence and predictive factors associated with immune-related liver injury and the relationship between irAE and treatment response were analyzed.

**Results:**

During a median of 10 (1–103) cycles with a median follow-up after several ICI initiations of 384 (21–1715) days, irAEs with all grades and with grade ≥ 3 developed in 144 (27.0%) and 57 (10.7%) cases. Cumulative irAE development rates were 21.9, 33.5, and 43.0% in all grades and 8.8, 14.9, and 20.7% in grade ≥ 3 at 5, 10, and 20 cycles, respectively. Patients who received anti-CTLA4 therapy were more likely to develop irAEs compared to those who received anti-PD-1 or anti-PD-L1 monotherapy. Liver injury was the most common irAE. Multivariate analysis identified the combination of PD-1 and anti-CTL-4 antibodies (hazard ratio [HR], 17.04; *P* < 0.0001) and baseline eosinophil count ≥130/μL (HR, 3.01 for < 130; *P* = 0.012) as independent risk factors for the incidence of immune-related liver injury with grade ≥ 2. Patients who developed irAEs had a higher disease control rate (*P* < 0.0001) and an increased overall survival rate compared to those without irAEs (*P* < 0.0001).

**Conclusion:**

Combination therapy with anti-PD-1 and anti-CTL-4 antibodies resulted in higher a frequency of irAEs. Baseline absolute eosinophil count was found to be a predictive factor for immune-related liver injury. Occurrence of irAEs may be associated with higher efficacy of ICI treatment and longer survival among patients who receive ICI therapy.

**Supplementary Information:**

The online version contains supplementary material available at 10.1186/s12885-022-10327-7.

## Introduction

Immune checkpoint inhibitors (ICIs), such as monoclonal antibodies (mAbs) against programed cell death 1 (PD-1), programmed cell death-ligand 1 (PD-L1) and cytotoxic T-lymphocyte antigen 4 (CTLA-4), have led to advances in cancer therapy, and ICI therapy has improved survival in patients with advanced-stage cancers. ICIs provide impressive anti-tumor activity in many solid tumors but are associated with immune-related adverse events (irAEs). ICI-induced irAEs develop as a consequence of impaired self-tolerance from loss of T-cell inhibition and may potentially damage many organs, including the lung, liver, gastrointestinal tract, skin and endocrine organs [1]. Although the mechanisms responsible for irAE remain unclear, some potential mechanisms include increasing T-cell activity against antigens that are present in tumors and healthy tissue, increased levels of preexisting autoantibodies, induction of inflammatory cytokines, and enhanced complement-mediated inflammation due to direct binding of an antibody against CTLA-4 expressed on normal tissue [1].

Recently, some studies have investigated risk factors associated with irAEs. A systematic review and meta-analysis, for example, has shown distinct patterns of irAEs according to the ICI class (CTLA-4 or PD-1/PD-L1) or tumor type (melanoma or non-melanoma) [2, 3]. However, in these studies, only the types of drugs were investigated as risk factors for irAEs.

The relationship between occurrence of irAEs and the treatment response to ICIs is gradually becoming clearer. Some retrospective studies have reported that occurrence of irAEs is associated with clinical benefits, such as better treatment responses or prognoses for certain malignancies [4–6]. Other studies have demonstrated that overall and progression-free survival were longer in patients who developed irAEs than in those who did not [7, 8]. In contrast, in a retrospective study of ipilimumab, the treatment outcomes were similar in patients with and those without irAEs [9].

In this study, we aimed to evaluate the prevalence and predictive factors associated with occurrence of irAEs, and the effect of irAEs on clinical outcomes in ICI-treated Japanese patients with malignancies.

## Materials and methods

### Patients

In total, 533 cases who were treated with ICIs, including anti-PD-1 (nivolumab or pembrolizumab), anti-PD-L1 (atezolizumab or durvalumab), or anti-CTLA-4 antibodies (ipilimumab), between September 2014 and January 2021 at Hiroshima University Hospital in Japan were enrolled in this retrospective observational study. Patients who were treated with ICIs in combination with another chemotherapy, such as a cytotoxic agent or molecular targeted agent, were not included in this study. Patients were excluded if they were < 18 years old. A baseline laboratory test was performed as per routine clinical practice that included blood count, kidney and liver function, and anti-nuclear antibody. After starting treatment, clinical and laboratory tests were carried out as clinically indicated every 2 or 3 weeks in patients receiving ICI therapy, prior to administration of the drug. Body computed tomography (CT) scans and other imaging tests, including magnetic resonance imaging (MRI) or positron emission-CT (PET-CT) were performed as clinically mandated. All patients were followed up until death or loss of contact. This study was carried out in accordance with the Declaration of Helsinki, and the Hiroshima University Hospital Institutional Ethics Committee approved this study. Informed consent was obtained in the form of opt-out on the website.

### Assessment of irAEs

We collected baseline characteristics, clinical outcomes, and adverse events from clinical records. Adverse events with a reasonable possibility of having an underlying immunological basis, including liver injury, interstitial pneumonia, hypothyroidism, rash, adrenal insufficiency, colitis and diarrhea or autoimmune diabetes had been diagnosed as irAEs by the attending physician. We evaluated irAEs on clinical records and graded irAEs using the Common Terminology Criteria for Adverse Events (CTCAE) version 5.0. The criteria for each irAE are shown in supplementary Table 1. Other adverse events such as infectious diseases, worsening of previous disorders, nausea, loss of appetite, etc., were not considered immune-mediated and were, therefore, not included in the present analysis.

### Statistical analysis

Categorical variables were assessed using the chi-squared test. Continuous variables are reported using the median and were assessed using the nonparametric Mann-Whitney U test. Factors associated with occurrence of immune-related liver injury with grade ≥ 2 were identified by the log-rank test. Cut-off values for continuous predictive factors were determined using receiver operating characteristic curves (ROC). Variables that achieved *P* < 0.05 in univariate analysis were entered into a Cox proportional hazards model. The overall survival rates among patients were calculated by the Kaplan–Meier method and compared with the log-rank test. The relationship between irAE development and overall survival were confirmed using the Cox proportional hazards regression model with the survival package in R version 4.2.0. (https://CRAN.R-project.org/package=survival). Other statistical analysis was performed using IBM SPSS version 22.0.0.0. Statistical significance was defined as having a *p*-value less than 0.05 (*P* < 0.05).

## Results

### Characteristics of patients

In this study, we identified 533 cases who had been treated with ICIs for advanced malignancies. Patient characteristics are summarized in Table 1. In total, 373 (70.0%) of the cases were male, and the median age was 67 (18–93) years. Patients had received a median of 10 (1–103) cycles of ICI therapy, with a median follow-up after several ICI initiations of 384 (21–1715) days. Overall, 20 patients (3.8%) had previously been exposed to another form of immunotherapy. The most common malignancies treated with ICIs were lung cancers (*n* = 209, 39.2%) and head-and-neck carcinomas (*n* = 106, 19.9%). Patients were treated with anti-PD-1 antibody (*n* = 452, 84.8%), anti-PD-L1 antibody (*n* = 44, 8.3%), anti-CTLA-4 antibody (*n* = 19, 3.6%), or a combination of anti-PD-1 and CTLA-4 antibodies (*n* = 18, 3.4%).

**Table 1:** Baseline characteristics of ICI-treated patients

	533 cases
Age (years)	67 (18–93)
Male/female	373/160
ECOG performance status, 0/1/≥2	313/173/47
Number of dosing cycles, median	10 (1–103)
Observation period, median (days)	384 (21–1715)
History of prior immunotherapy	20
Comorbidity	
Hypertension	152
Diabetes	89
Liver disease	39
Thyroid disorders	37
Tumor type	
Lung cancer	209
Head-and-neck cancer	106
Malignant melanoma	50
Urothelial cancer	60
Gastric cancer	36
Esophageal cancer	27
Malignant mesothelioma	26
Others	19
Immune checkpoint therapy	
Anti-PD-1 antibody	452
Nivolumab/pembrolizumab	328/124
Anti-PD-L1 antibody	44
Atezolizumab/durvalumab	32/12
Anti-CTLA-4 antibody (ipilimumab)	19
Combination of anti-PD-1 and CTLA-4 antibodies	18
Nivolumab and ipilimumab	18

*ICI* immune checkpoint inhibitor, *PD-1* programmed death 1, *PD-L1* programmed death-ligand 1, *CTLA-4* cytotoxic T-lymphocyte antigen 4

### Prevalence of irAEs

**Fig. 1:**
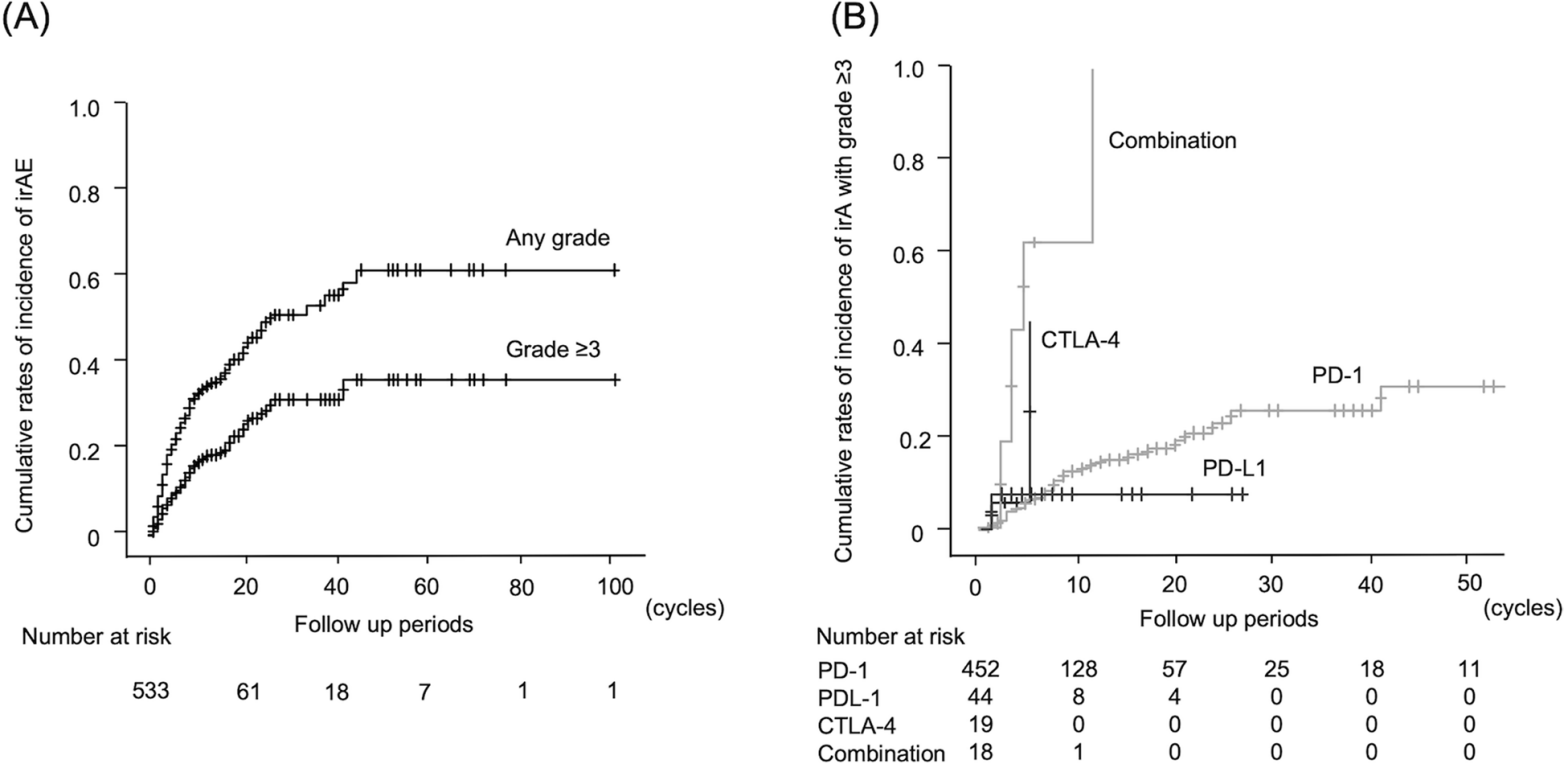
Cumulative rates of development immune-related adverse events (irAEs) in patients with malignancy who were treated with immune checkpoint inhibitors. **a** Cumulative development rates with any grade and grade ≥ 3 of irAEs. **b** Cumulative development rates with grade ≥ 3 of irAEs according to the regimen

During the observation period, irAEs of any grade or with grade ≥ 3 developed in 144 (27.0%) and 57 (10.7%) cases, respectively (Table 2). The most common irAEs were liver injury (35 cases [6.6%]), hypothyroidism (33 cases [6.2%]), interstitial pneumonia (32 cases [6.0%]), rash (21 cases [3.9%]), adrenal insufficiency (14 cases [2.6%]), colitis and diarrhea (11 cases [2.1%]), autoimmune diabetes (2 cases [0.4%]), and other (20 cases [3.8%]). Cumulative irAE development rates were 21.9, 33.5, and 43.0%, for any grade, and 8.8, 14.9, and 20.7% for grade ≥ 3 at 5, 10, and 20 cycles, respectively (Fig. 1A). With respect to type of ICI therapy, irAEs of any grade and with grade ≥ 3 developed in 114 (25.2%) and 42 (9.3%) cases with anti-PD-1 therapy, 7 (15.9%) and 3 (6.8%) cases with anti-PD-L1 therapy, 9 (47.4%) and 3 (15.8%) cases with anti-CTLA-4 therapy, and 14 (77.8%) and 9 (50.0%) cases for the combination of anti-PD-1/PD-L1 and anti-CTL-4 therapies, respectively (Table 2). Patients who received anti-CTLA4 therapy were more likely to develop irAEs compared to those who received anti-PD-1 or anti-PD-L1 monotherapy. Cumulative rates of irAEs with grade ≥ 3 were higher in patients who received anti-CTLA4 therapy compared to those who received anti-PD-1 or anti-PD-L1 monotherapy (*P* < 0.001; anti-CTLA-4 with or without anti-PD-1 vs anti-PD-1 or anti-PD-L1 monotherapy) (Fig. 1B).

**Table 2:** Frequencies of immune-related adverse events (irAEs)

	All cases		Anti-PD-1 antibody		Anti-PD-L1 antibody		Anti-CTLA-4 antibody		Combination of anti-PD-1 and CTLA-4 antibodies	
N (%)	533 cases		452 cases		44 cases		19 cases		18 cases	
	All grade	Grade ≥ 3	All grade	Grade ≥ 3	All grade	Grade ≥ 3	All grade	Grade ≥ 3	All grade	Grade ≥ 3
All events	144 (27.0)	57 (10.7)	114 (25.2)	42 (9.3)	7 (15.9)	3 (6.8)	9 (47.4)	3 (15.8)	14 (77.8)	9 (50.0)
Liver injury	35 (6.6)	19 (3.6)	24 (5.3)	12 (2.7)	2 (4.5)	0	1 (5.03	0	8 (44.4)	7 (38.9)
Interstitial pneumonia	32 (6.0)	14 (2.6)	31 (6.9)	13 (2.9)	1 (2.3)	1 (2.3)	0	0	0	0
Hypothyroidism	33 (6.2)	2 (0.4)	25 (5.5)	1 (0.2)	2 (4.5)	0	3 (15.8)	0	3 (16.7)	1 (5.6)
Rash	21 (3.9)	2 (0.4)	16 (3.5)	2 (0.4)	0	0	1 (5.3)	0	4 (22.2)	0
Adrenal insufficiency	14 (2.6)	6 (1.1)	10 (2.2)	4 (0.9)	0	0	2 (10.5)	1 (5.3)	2 (11.1)	1 (5.6)
Colitis and diarrhea	11 (2.1)	3 (0.6)	9 (2.0)	3 (0.6)	0	0	1 (5.3)	0	1 (5.6)	1 (5.6)
Autoimmune diabetes	2 (0.4)	2 (0.4)	2 (0.4)	2 (0.4)	0	0	0	0	0	0
Others	20 (3.8)	14 (2.6)	11 (2.4)	9 (2.0)	3 (6.8)	2 (4.5)	3 (15.8)	2 (10.5)	3 (16.7)	2 (11.1)

*PD-1* programmed death 1, *PD-L1* programmed death-ligand 1, *CTLA-4* cytotoxic T-lymphocyte antigen 4

### Prevalence and risk factors associated with the incidence of immune-related liver injury

We focused on liver injury, the most common irAE in this study. During the observation period, 35 (6.6%) and 19 (3.6) patients developed immune-related liver injury with any grade or grade ≥ 3, and this was the highest frequency of any irAE (Table 2). Table 3 shows the characteristics and treatment regimens in 35 patients with immune-related liver injury. Among the 28 patients with liver injury of grade ≥ 2, 16 (57.1%), 10 (35.7%), and 2 (7.2%) patients developed hepatocellular-type, cholestatic-type and mixed-type liver injury, respectively. Histopathological analysis of liver biopsy specimens was performed in 7 patients. Patients with immune-related liver injury of grade ≥ 2 had undergone careful liver function monitoring, and patients with grade ≥ 3 had discontinued ICI therapy and were treated instead with prednisolone (PSL) as an alternative treatment strategy. PSL was not administered to the four cases with grade 3 who improved quickly with ICI withdrawal or administration of ursodeoxycholic acid (UDCA) alone, while three patients with grade 2 were treated with PSL because of prolonged liver dysfunction and other irAE complications. Eighteen patients were administered PSL, and 6 patients were treated with UDCA. Three out of 18 patients who received PSL therapy were subsequently treated with mycophenolate mofetil (MMF) due to lack of improvement.

**Table 3:** Patients with immune-related liver injury

35 patients	n (%)
CTCAE grade (n)	
1/2/3/4/5	7/9/14/4/1
Tumor type (n)	
Hep/Chol/Mix (grade ≥ 2)	16/10/2
Liver biopsy	7 (20.0)
Anti-PD-1 antibody	6
Combination therapy	1
Treatment	
Prednisolone	18 (51.4)
Ursodeoxycholic acid	6 (17.1)
Mycophenolate mofetil	3 (8.6)
Withdrawal of ICIs	26 (74.3)

*PD-1* programmed death 1, *Hep* Hepatocellular-type, *Chol* cholestatic-type, *Mix* mixed-type

We next evaluated factors associated with the incidence of immune-related liver injury. Univariate analysis showed that history of prior immunotherapy, the use of anti-PD-1 antibodies, the combination of anti-PD-1 and anti-CTL-4 therapy, and baseline lymphocyte and eosinophil counts were significantly associated with incidence of liver injury with grade ≥ 2 (Table 4). When the optimal cutoff value was determined by ROC analysis, multivariate analysis identified the combination of PD-1 and anti-CTL-4 antibodies (hazard ratio [HR], 17.04; *P* < 0.0001) and baseline eosinophil count ≥130/μL (HR, 3.01 for < 130; *P* = 0.012) as independent predictive factors for the incidence of immune-related liver injury.

**Table 4:** Factors associated with immune-related liver injury with grade ≥ 2 development

	Incidence of irAE		Univariate analysis	Multivariate analysis	
Factors	with	without	*P* value	Hazard ratio (95% CI)	*P* value
Age, years < 69/69≤	12/16	254/251	0.34		
Male/female	16/12	357/148	0.16		
ECOG performance status, 0 or 1/≥2	28/0	458/47	0.19		
History of prior immunotherapy, yes/no	3/25	17/493	0.015		
Hypertension, yes/no	12/16	130/365	0.12		
Diabetes, yes/no	5/23	84/421	0.84		
Liver disease, yes/no	0/28	39/466	0.10		
Thyroid disorders, yes/no	0/28	37/468	0.11		
Anti-PD-1 antibody, with/without	17/11	435/70	< 0.0001		
Anti-PD-L1 antibody, with/without	2/26	42/463	0.99		
Anti-CTLA-4 antibody, with/without	1/27	18/487	0.47		
Combination of anti-PD-1/PDL-1 and anti-CTLA-4, with/without	8/20	10/495	< 0.0001	17.04 (7.07–41.08)	< 0.0001
Neutrophil count, < 4050/4050≤/μL	13/15	254/248	0.29		
Lymphocyte count, < 1175/1175≤/μL	8/20	264/238	0.013		
Eosinophil count, < 130/130≤/μL	7/21	274/228	0.003	3.01 (1.27–7.12)	0.012
Hemoglobin, < 11.6/11.6 ≤ g/dL	8/20	257/243	0.08		
Platelet count, < 238/238 ≤ ×10^3^/μL	12/14	247/215	0.18		
Prothrombin activity, < 91/91 ≤ %	2/6	67/68	0.29		
Total bilirubin, < 0.5/0.5 ≤ mg/dL	9/18	159/334	0.69		
Aspartate aminotransferase, < 22/22 ≤ U/L	12/16	243/257	0.58		
Alanine aminotransferase, < 15/≥15 ≤ U/L	10/18	233/267	0.19		
Alkaline phosphatase, < 254/254 ≤ U/L	12/15	203/206	0.29		
γ-glutamyltransferase, < 30/30 ≤ U/L	15/9	214/230	0.40		
Lactate dehydrogenase, < 200/200 ≤ U/L	17/11	278/228	0.97		
Albumin, < 3.7/3.7 ≤ g/dL	10/11	226/225	0.75		
Creatinine, < 0.80/0.80 ≤ mg/dL	14/14	243/254	0.49		
Estimated glomerular filtration rate, < 69/69 ≤ mL/min/1.73m^2^	16/12	248/249	0.62		
C-reactive protein, < 0.79/0.79 ≤ mg/dL	11/12	201/211	0.49		
Fasting blood sugar, < 105/105 ≤ mg/dL	11/6	138/151	0.22		
Hemoglobin A1c, < 5.8/5.8 ≤ %	9/6	118/145	0.36		
Antinuclear antibodies, <×80/×80≤	9/1	109/16	0.98		

*PD-1* programmed death 1, *PD-L1* programmed death-ligand 1, *CTLA-4* cytotoxic T-lymphocyte antigen 4

### Association of irAEs with treatment efficacy

**Fig. 2:**
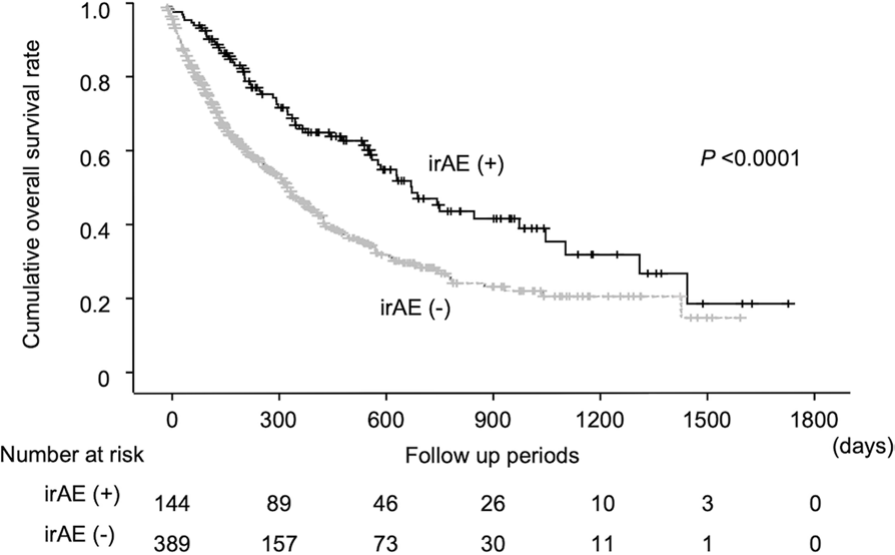
Cumulative overall survival rates according to incidence of immune-related adverse events (irAEs). Overall survival rate was significantly higher in patients who developed irAEs during immune checkpoint inhibitor treatment

Among the 533 patients, treatment response could be judged in 479 cases, but 54 cases remained undetermined due to the short duration of the observation period. In all patients, disease control was observed in 153 cases (31.9%); complete response (CR) in 15 cases (3.1%), partial response (PR) in 52 cases (10.9%) and stable disease (SD) in 86 cases (18.0%), while progressive disease was detected in 326 cases (68.1%). Disease control (CR, PR plus SD) was achieved in 66 out of 129 cases (51.2%) with irAEs and 87 out of 350 cases (24.9%) without (*P* < 0.0001). The median number of cycles of ICI therapy that were received was not different between patients with and without irAEs (6 [1–69] and 5 [1–103], respectively), suggesting that patients who developed irAEs were more likely to experience greater effectiveness of ICI therapy compared to patients who did not develop irAEs. Overall survival curves estimated with the Kaplan-Meier method based on the presence or absence of any irAE showed that the development of irAEs was significantly associated with a longer survival (*P* < 0.0001) (Fig. 2). Cox proportional hazards regression also confirmed that irAE development and ECOG performance status were significantly associated with overall survival when accounting for other factors such as age, sex, ICI treatment type, and tumor type (Supplementary Table 2). When overall survival was analyzed with respect to tumor type, patients with irAEs had significantly longer survival than those without irAE in both lung cancer, which comprised the majority of malignancies in this study (*P* = 0.001), as well as in other non-lung cancer malignancies (*P* = 0.006).

## Discussion

We observed an overall irAE incidence of 27.0% across all cancer types considered. This irAE frequency is similar to that of a recent study based on 16 RCT studies in which the incidence of irAEs of all grades and for grade ≥ 3 was estimated to affect 35.6 and 7.3% of patients, respectively, for lung cancer treated with ICIs [3].

Occurrence of irAEs was especially common among patients who received anti-CTLA therapy, and multivariate analysis identified the use of the combination of anti-PD-1 and anti-CTL-4 antibodies as an independent risk factor for the incidence of immune-related liver injury with grade ≥ 2. The combination of anti-PD-1 and anti-CTL-4 therapy accounted for 77.8% of irAEs of any grade and 50.0% of irAEs with grade ≥ 3, respectively. These results are similar to previous reports that showed an increased incidence of irAEs in the case of ICI combination therapies [10–12]. Based on previous reports, immune-related liver injury is estimated to affect 1–4% and 4–9% of patients treated with anti-PD-1/PD-L1 and CTLA-4 antibody alone, respectively, and 18% of patients treated with the combination of anti-PD-1 and anti-CTLA-4 antibody [13–15]. Moreover, in a study of combination therapy with anti-PD-1 and anti-CTLA-4 for metastatic melanoma, 37% of patients developed irAE liver injury of any grade, and 16% reported grade ≥ 3 [16]. Because the anti-tumor effects of anti-PD1/PD-L1 and anti-CTLA-4 employ different mechanisms, the combination of these ICIs promises higher potency against various malignancies but also increases the risk of irAEs.

Because the mechanisms of irAEs differ by organ, we focused on analyzing the characteristics and risk factors for liver injury, which was the most common irAE. Among the 28 patients with grade ≥ 2 immune-related liver injury in this study, 16 (57.1%) developed hepatocellular-type liver injury, 10 (35.7%) developed cholestatic-type liver injury, and the remaining 2 patients (7.1%) developed mixed-type liver injury, as assessed by CTCAE. The distribution of ICI-induced liver injury is similar to a previous report that analyzed 56 patients with ICI-induced liver injury [17]. The current recommendation for Grade 2 liver injury is discontinuation of ICIs and switching to corticosteroid therapy at a dose of 0.5–1.0 mg/kg/day. When patients develop Grade 3–4 liver injury, corticosteroid therapy at a dose of 1.0–2.0 mg/kg/day is used. If liver injury fails to respond to corticosteroid therapy, addition of MMF is a fallback treatment option [18]. In the present study, among the 18 patients who received PSL therapy for ICI-induced liver injury, 3 patients were also treated with MMF, but two patients died.

We performed histopathological analysis of liver biopsy on 7 patients with immune-related liver injury of grade ≥ 3. Immunostaining revealed predominantly CD8-positive T cell infiltration into the liver, and, to a lesser extent, CD4-positive and CD20-positive T cells in all patients (Fig. S1). Pathological findings are diagnostic in immune-related liver injury. The most common patterns are acute hepatitis with spotty or confluent lobular inflammation and centrilobular necrosis [19, 20]. In a previous report, immunostaining revealed that the majority of lymphocytes involved in both periportal and lobular inflammatory infiltration were CD8-positive lymphocytes [20, 21]. Zen et al. compared the histological features of patients with drug-induced liver injury, autoimmune hepatitis, and ICI-induced immune-mediated liver injury and found that immunostaining revealed the presence of large numbers of CD8-positive T cells, whereas CD20-positive B cells and CD4-positive T cells were fewer in ICI-induced liver injury than in drug-induced liver injury or autoimmune hepatitis or drug-induced liver injury [20]. In fact, the present study also showed that infiltration of lymphocytes in the liver is mainly CD8-positive T cells. The relationship between the proportion of infiltrating lymphocytes and the pathological conditions in immune-related liver injury is unclear. Only seven patients underwent liver biopsy examination in this study, but it would be interesting to analyze the correlation between the proportions of CD4+, CD8+, CD20, or FOXP3-positive T cells and pathological conditions such as the diagnosis, severity and treatment response of irAEs using a larger number of histological examinations in the future.

The present study showed that a higher baseline white blood cell count including lymphocytes and eosinophils and a baseline eosinophilic count ≥130/μL were independent risk factors for the incidence of immune-related liver injury of grade ≥ 2. Several host factors have been reported to be associated with the occurrence of irAEs including female sex, baseline absolute lymphocyte and eosinophil numbers, presence of autoantibodies, sarcopenia, fever within 24 hrs of initial ICI administration, composition of the gut microbiome, and elevated BMI [22–27]. Diehi et al. demonstrated that higher baseline absolute lymphocyte number was an independent risk factor for irAEs of grade ≥ 2 for anti-PD-1 antibody-treated patients with solid tumors [23]. Chu et al. reported that a higher baseline absolute eosinophil count was associated with the occurrence of ICI-induced pneumonia [26], and Nakamura et al. reported that a higher baseline absolute eosinophil count was associated with the occurrence of endocrine irAEs [22]. Eosinophils play a role in regulating multiple immune functions, such as activation of T cells by antigen presentation and attraction of tumor-specific CD8-positive T cells [26]. Both lymphocytes and eosinophils are important for immunity; therefore, the number of white blood cells might correlate with the occurrence of irAEs. These mechanisms of immune response may be involved in the development of irAEs. Although the specific predictive factors for ICI-induced liver injury are unclear, the results of this study may be useful for identifying early-onset irAEs, considering the availability of blood cell count data prior to the initiation of ICI treatment.

The present study showed a higher response rate to ICI therapy and an increased overall survival rate in patients who developed irAEs. The relationship between occurrence of irAEs and treatment response to ICIs remains controversial. Some studies have shown clinical benefits such as better treatment response or prognosis [4–8, 28, 29], but other large retrospective studies of ipilimumab have shown similar treatment outcomes between patients whether or not they experienced irAEs [9]. ICIs target not only tumor-specific T cells but also other T cells and may cause the unintended activation of non-tumor-specific T cells, resulting in irAEs in a variety of organs. Recent studies suggest another possible mechanism underlying irAEs. T cells that target antigens common to both tumors and healthy tissue are activated by ICIs, leading both to higher antitumor efficacy as well as a higher incidence of irAEs [30]. This mode of T cell activation might lead to irAEs in various organs while at the same time enhancing the anti-tumor effect.

This study has several limitations due to its retrospective nature. First, no clear diagnostic criteria for irAEs could be used; irAEs were diagnosed based on symptoms or laboratory tests temporally associated with the use of ICIs. Although irAEs were diagnosed by the attending physician and carefully evaluated retrospectively by us based on medical records, some irAEs, especially mild non-hematological irAEs, such as colitis or cholangitis, might have been overlooked; however, we believe that most severe irAEs were detected. We determined irAE grade ≥ 2 as significant in this study. Second, a variety of malignancies and treatment regimens were analyzed together in this study even though the number of patients with some malignancies was small. The pathological finding of irAEs might vary by cancer type. It may be necessary to identify the specific predictive factors associated with occurrence of specific irAEs and clarify the influence of irAE development on prognosis with respect to malignancy and treatment regimen. Third, several important pieces of clinical information were lacking, including details on concomitant medications that could induce adverse events. Although we confirmed based on the medical records that there was no significant change in concomitant medications used during the course of ICI therapy, it is possible that some adverse events observed in this study were caused by concomitant medications rather than as a direct result of ICI therapy. Lastly, the present study showed that the development of irAEs was associated with a higher response rate to ICI therapy and an increased survival rate. Although the median number of cycles of ICI therapy that were received was not different between patients with and without irAEs, it is not possible to exclude the possibility that longer treatment periods increased the chance of irAE development. To reduce this bias, the relationship between irAEs and survival was analyzed by Cox proportional hazards regression model, and the model showed that the presence of irAEs was significantly associated with survival after controlling for factors such as age, cancer type, and performance status. This result seems to support the conjecture that the presence of irAEs is associated with improved survival, but further analysis is needed to clarify the predictive factors for occurrence of irAEs and the relationship between the incidence of irAEs, treatment response to ICI therapy, and survival for each type of therapy in a prospective multicenter study.

In conclusion, we conducted a retrospective study on patients with malignancies who received ICI therapies such as anti-PD-1, PD-L1, and CTLA-4 antibodies. Patients who received anti-CTLA4 therapy were more likely to develop irAEs compared to those who received anti-PD-1 or anti-PD-L1 monotherapy. Use of ICI combination therapies, such as anti-PD-1 and anti-CTLA-4 antibodies, and baseline absolute eosinophil count might be predictive factors for the occurrence of immune-related liver injury, the most common irAE. The occurrence of irAEs seems to be associated with a higher efficacy of ICI therapy as well as longer survival.

## Supplementary Information

**Additional file 1: Supplementary Table 1.** Criteria for diagnosis or relationship of each irAE. **Supplementary Table 2.** Cox proportional hazards regression model for overall survival in patients treated with immune checkpoint inhibitors. **Fig. S1.** Histological analysis of the liver. Liver samples in each of seven patients were stained with either hematoxylin-eosine (HE) or immunostained with anti-CD4, anti-CD8, and anti-CD20 antibodies (original magnification, × 200). Arrowheads indicate CD8-positive cells.

## Data Availability

All data generated or analyzed during this study are included in this published article and its supplementary information files.

## Abbreviations

CTACE: Common Terminology Criteria for Adverse Events
irAE: Immune-related adverse events
ICIs: Immune checkpoint inhibitors
PD-1: Programmed death 1
PD-L1: Programmed death-ligand 1
CTLA-4: Cytotoxic T-lymphocyte antigen 4
CI: Confidence interval
HR: Hazard ratio
MMF: Mycophenolate mofetil
ROC: Receiver operating characteristic curves
PSL: Prednisolone
UDCA: Ursodeoxycholic acid
