# Supplementary material for: Prevalence of immune-related adverse events and anti-tumor efficacy following immune checkpoint inhibitor therapy in Japanese patients with various solid tumors
Source: BMC Cancer. 2022 Nov 29;22:1232. doi: 10.1186/s12885-022-10327-7 (PMC9706984; doi:10.1186/s12885-022-10327-7)
Supplement: Supplementary file 1 — Additional file 1: Supplementary Table 1. Criteria for diagnosis or relationship of each irAE. Supplementary Table 2. Cox proportional hazards regression model for overall survival in patients treated with immune checkpoint inhibitors. Fig. S1. Histological analysis of the liver. Liver samples in each of seven patients were stained with either hematoxylin-eosine (HE) or immunostained with anti-CD4, anti-CD8, and anti-CD20 antibodies (original magnification, × 200). Arrowheads indicate CD8-positive cells. [file 12885_2022_10327_MOESM1_ESM.docx]

**Supplementary table 1. Criteria for diagnosis or relationship of each irAE.**

| Event | Criteria and clinical parameters |
| --- | --- |
| Liver injury | Blood tests showing abnormal levels of ALT, AST, ALP or T-Bil and liver injury with a reasonable possibility of having an underlying immunological basis were diagnosed as irAEs. Patients with other causes of liver injury, including other drugs, nutrition disorders, biliary obstruction, or exacerbation of liver metastasis were excluded. |
| Interstitial pneumonia | The diagnosis was made by excluding non-ICI causes of pneumonitis, such as infection or malignancy when respiratory symptoms or decreased SpO2 are present and CT or chest x-ray showed imaging findings such as infiltrating shadows with reference to KL-6 and SP-D values. This may require consultation from infectious disease and pulmonology specialists. |
| Hypothyroidism | Diagnosed by abnormal endocrine function tests (through thyroid-stimulating hormone (TSH) and free thyroxine (T4) tests). Occasionally, endocrine consultation should be considered in cases of symptomatic hyperthyroidism to ensure resolution of symptoms. |
| Rash | Skin rash or itching is diagnosed by the attending physician as being related to ICI administration. If there is an exacerbation or atypical presentation, a dermatologist is consulted and the patient is evaluated for alternative etiologies (e.g., infection or another systemic illness) with consideration of biopsy. |
| Adrenal insufficiency | Adrenal insufficiency could be diagnosed by biochemical testing (TSH, luteinizing hormone, follicle stimulating hormone, adrenocorticotropic hormone, cortisol, and testosterone/estrogen levels) by an endocrinologist when subjective symptoms such as fatigue, black mouth are present. |
| Colitis and diarrhea | Diagnosed by the attending physician when symptoms of diarrhea (4 or more times per day or persistent watery stools), abdominal pain, or bloody stools were present or when CT scans showed evidence such as bowel wall thickening and fluid-filled colonic distension. GI consultation and endoscopic evaluation are recommended for suspected cases of ≥ grade 2 toxicity as it confirms the diagnosis and determines the extent and severity of the inflammation. |
| Autoimmune diabetes | If a patient develops new-onset hyperglycemia, additional testing of C-peptide levels and testing for the presence of glutamic acid decarboxylase (GAD) can differentiate between T1DM and T2DM. Diagnosis is made by an endocrinologist based on laboratory findings (test for hemoglobin A1C, blood gas, urine ketones, and serum hydroxybutyrate levels) and clinical symptoms. |
| Others | Immunological adverse events diagnosed by the attending physician or specialists that were thought to be related to ICI administration, such as myositis, renal failure, and pancreatitis. |

irAEs were graded based on the Common Terminology Criteria for Adverse Events version 5.0.

**Supplementary table 2. Cox proportional hazards regression model for overall survival in patients treated with immune checkpoint inhibitors.**

| Factor | coef | exp(coef) | se(coef) | z | Pr(>\|z\|) |  |
| --- | --- | --- | --- | --- | --- | --- |
| irAE | -0.54 | 0.58 | 0.16 | -3.46 | 0.000541 | *** |
| Age >69 | 0.02 | 1.02 | 0.13 | 0.14 | 0.892 |  |
| Sex | 0.18 | 1.19 | 0.14 | 1.26 | 0.209 |  |
| Prior ICI therapy | 0.26 | 1.29 | 0.40 | 0.63 | 0.528 |  |
| ECOG performance status >2 | 0.87 | 2.39 | 0.20 | 4.39 | 1.15E-05 | *** |
| Comorbidity |  |  |  |  |  |  |
| Diabetes | -0.03 | 0.98 | 0.17 | -0.15 | 0.883 |  |
| Hypertension | -0.20 | 0.82 | 0.15 | -1.30 | 0.193 |  |
| Thyroid disorder | 0.16 | 1.18 | 0.24 | 0.68 | 0.496 |  |
| Liver disease | -0.15 | 0.86 | 0.25 | -0.59 | 0.558 |  |
| Autoimmune disease | 0.38 | 1.46 | 0.42 | 0.89 | 0.375 |  |
| Tumor type |  |  |  |  |  |  |
| Lung cancer | 0.10 | 1.10 | 0.37 | 0.25 | 0.799 |  |
| Malignant melanoma | -0.02 | 0.98 | 0.42 | -0.04 | 0.971 |  |
| Urothelial cancer | -0.32 | 0.73 | 0.42 | -0.76 | 0.446 |  |
| Head and neck cancer | 0.03 | 1.04 | 0.38 | 0.09 | 0.928 |  |
| Gastric cancer | 0.28 | 1.32 | 0.42 | 0.66 | 0.508 |  |
| Esophageal cancer | 0.43 | 1.54 | 0.47 | 0.91 | 0.363 |  |
| Malignant mesothelioma | 0.15 | 1.16 | 0.47 | 0.32 | 0.752 |  |
| Immune checkpoint therapy |  |  |  |  |  |  |
| Anti-CTLA-4 antibody | 0.43 | 1.54 | 0.41 | 1.05 | 0.296 |  |
| Anti-PD-1 and CTLA-4 antibodies | 0.18 | 1.20 | 0.50 | 0.36 | 0.716 |  |
| Anti-PD1 antibody | 0.24 | 1.28 | 0.28 | 0.86 | 0.388 |  |

ICI; immune checkpoint inhibitor, PD-1; programmed death 1, PD-L1; programmed death-ligand 1, CTLA-4; cytotoxic T-lymphocyte antigen 4

**Supplementary figure 1**

**
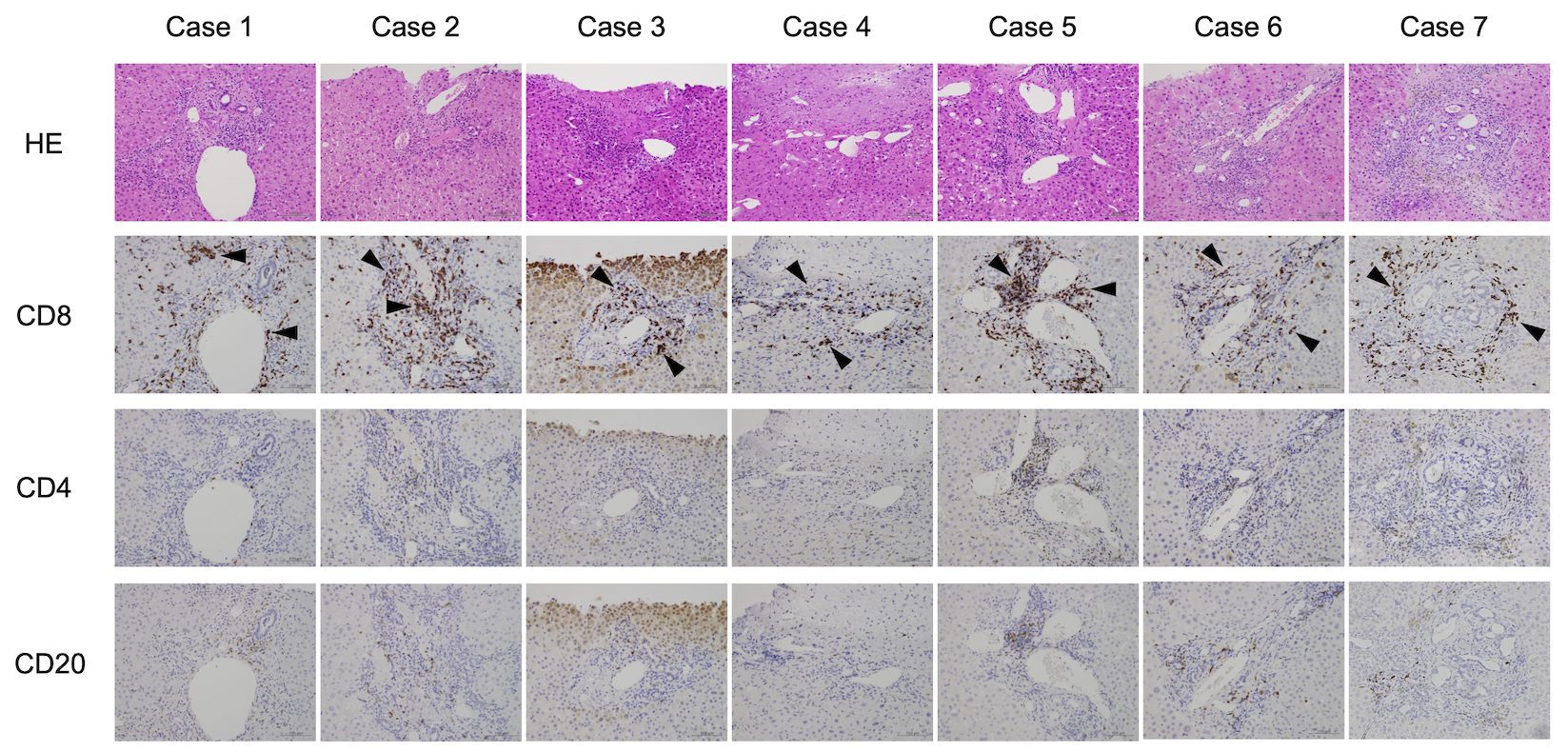
**

**Figure S1.** Histological analysis of the liver. Liver samples in each of seven patients were stained with either hematoxylin-eosine (HE) or immunostained with anti-CD4, anti-CD8, and anti-CD20 antibodies (original magnification, ×200). Arrowheads indicate CD8-positive cells.
